# Activity of xyloglucan endotransglucosylases/hydrolases suggests a role during host invasion by the parasitic plant *Cuscuta reflexa*

**DOI:** 10.1371/journal.pone.0176754

**Published:** 2017-04-27

**Authors:** Stian Olsen, Kirsten Krause

**Affiliations:** Department of Arctic and Marine Biology, Faculty of Biosciences, Fisheries and Economics, UiT The Arctic University of Norway, Tromsø, Norway; Universiteit Gent, BELGIUM

## Abstract

The parasitic vines of the genus *Cuscuta* form haustoria that grow into other plants and connect with their vascular system, thus allowing the parasite to feed on its host. A major obstacle that meets the infection organ as it penetrates the host tissue is the rigid plant cell wall. In the present study, we examined the activity of xyloglucan endotransglucosylases/hydrolases (XTHs) during the host-invasive growth of the haustorium. The level of xyloglucan endotransglucosylation (XET) activity was found to peak at the penetrating stage of *Cuscuta reflexa* on its host *Pelargonium zonale*. *In vivo* colocalization of XET activity and donor substrate demonstrated XET activity at the border between host and parasite. A test for secretion of XET-active enzymes from haustoria of *C*. *reflexa* corroborated this and further indicated that the xyloglucan-modifying enzymes originated from the parasite. A known inhibitor of XET, Coomassie Brilliant Blue R250, was shown to reduce the level of XET in penetrating haustoria of *C*. *reflexa*. Moreover, the coating of *P*. *zonale* petioles with the inhibitor compound lowered the number of successful haustorial invasions of this otherwise compatible host plant. The presented data indicate that the activity of *Cuscuta* XTHs at the host-parasite interface is essential to penetration of host plant tissue.

## Introduction

In order to sustain their own growth, parasitic plants infect other plants and absorb their nutrients. This parasitization is accomplished by the development of the haustorium, a specialized infection organ that is able to grow into the tissue of a compatible host plant and transfer water, minerals and sugars between the two plants [1]. Species of the holoparasitic genus *Cuscuta* are thread-like vines that coil around potential hosts and produce haustoria to infect the aboveground parts of plants [2]. Although the precise mechanisms of *Cuscuta* host infection remain enigmatic, evidence points to cell wall changes being central [3–6]. The current knowledge about plant attacks by other parasitic plant genera as well as by plant pathogens in general also argues for the significance of the cell wall [7, 8], indicating that plant invaders in general modify or degrade their host’s walls as a means of entry. The plant primary cell wall is built up of cellulose microfibrils, hemicelluloses, pectins and structural proteins, together constituting a boundary that has to be overcome before the haustorium can reach the host’s xylem and phloem and fuse with these vascular elements. Fortunately for the parasite, the plant cell wall is not a permanently closed border, but a complex dynamic structure that is continually being modified in order to regulate growth and development [9, 10]. Host infection by *Cuscuta* can be divided into three stages: the initial swelling of the parasite stem, the penetrating stage when the haustorium grows through the host tissue and the final mature stage when a connection has been established and the parasite is feeding on its host. We recently reported that *Cuscuta* genes encoding xyloglucan endotransglucosylases/hydrolases (XTHs) were highly expressed during the swelling stage of haustorium development [6]. These cell wall enzymes act on the hemicellulose xyloglucan, employing either one or both of two distinct mechanisms: xyloglucan endotransglucosylation (XET) and xyloglucan endohydrolysis (XEH) [11]. Through their activities of cutting and pasting xyloglucan, XTHs loosen or strengthen cell walls and are generally associated with the regulation of plant growth [12–14]. However, the diverse functionality of these enzymes is demonstrated by the fact that in land plants they are present in large gene families whose members display differing expression patterns [15–17].

To address the potential role of XTHs in *Cuscuta* parasitization, we analysed the level of xyloglucan endotransglucosylation and the *in vivo* colocalization of XET activity and donor substrate during infection of the compatible host *Pelargonium zonale*. The high XET activity observed at the host-parasite interface prompted us to test whether XTHs were secreted from haustoria of *Cuscuta reflexa*. To avoid contamination of XTHs from the host plant, host-independent haustoriogenesis was activated by far-red light and tactile stimuli [18] for the secretion tests. Finally, in order to investigate the necessity of XET activity for successful parasitization, we tested the effect of an XET inhibitor, Coomassie Brilliant Blue R250 (BB-R250), on *Cuscuta* host infection.

## Results

### XET activity during host infection

**Fig 1:**
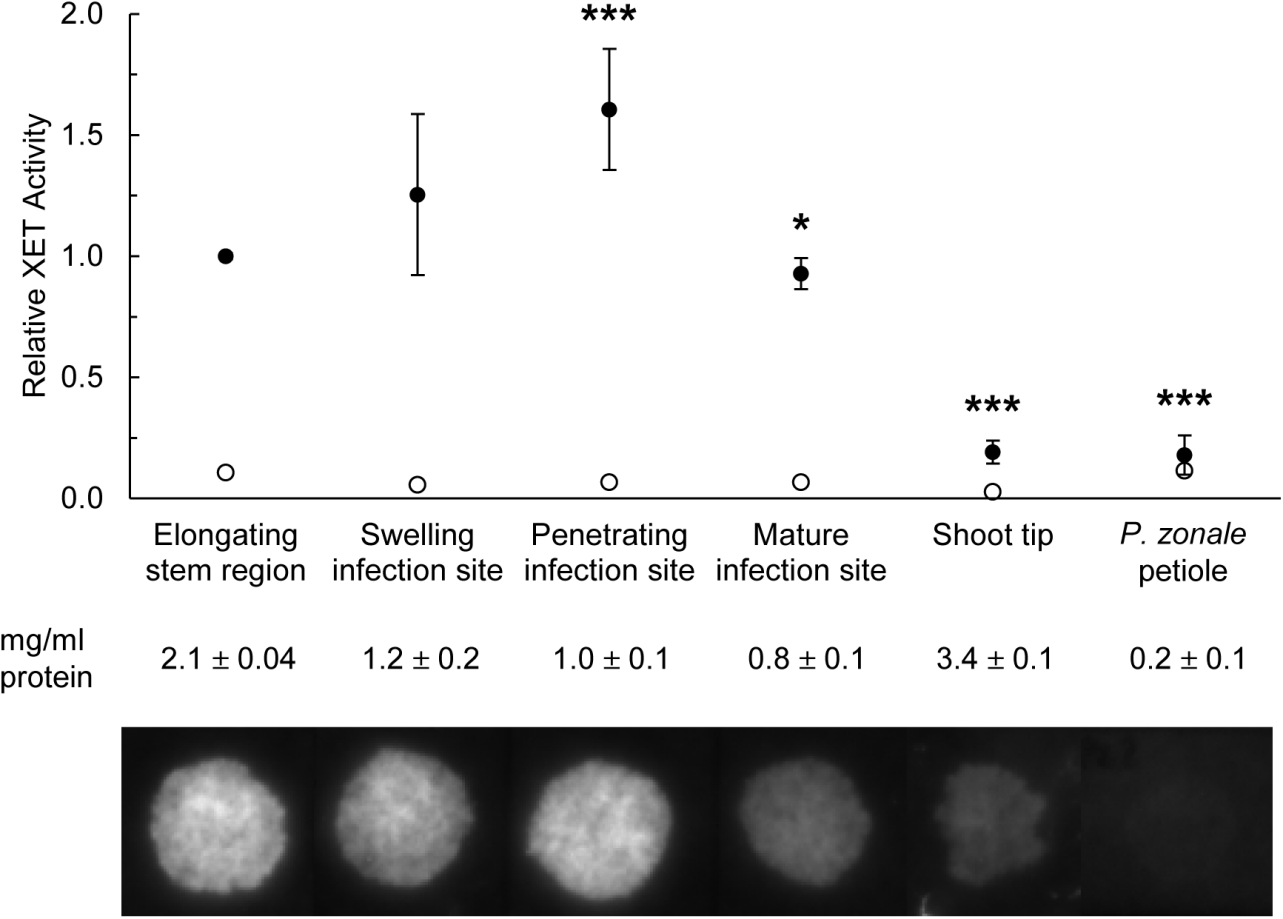
XET activity levels in developing haustoria of *C*. *reflexa*. Activity was measured as the incorporation of XyGO-SR into xyloglucan (exemplary fluorescence dot blot is shown below the chart). In the chart, fluorescence intensities (means ± standard deviation, n = 6 biological replicates for swelling, penetrating and mature infection sites and 3 biological replicates for elongating stem region, shoot tip and *P*. *zonale* petiole) are normalized to the concentration of total protein and presented in relation to the mean XET activity in elongating stem region (set to 1). Average protein concentrations ± standard deviations are shown below the chart. Statistically significant differences between the elongating stem region control and the other samples were calculated by an unpaired, two-tailed *t*-test assuming unequal variances and are indicated by asterisks (*, P < 0.05 and ***, P < 0.005). Empty circles indicate activity levels in boiled extracts.

In order to compare the levels of XET activity at different stages of haustorium development in *C*. *reflexa*, enzyme extracts were made from the parasite part of swelling, penetrating and mature infections sites on the host plant *P*. *zonale*. Enzyme extracts from shoot tips and elongating stem regions of the parasite and non-infected petioles of *P*. *zonale* were included as controls. Dot blot analysis showed that the XET activity normalized to the total protein concentration was highest when the parasite is committed to penetrating the host tissue (Fig 1). No fluorescence was detected when dot blotting on control papers. An incubation of the enzyme extracts for 10 min at 99°C eliminated the XET activity and confirmed that the activity is derived from heat-sensitive proteins in the extract.

**Fig 2:**
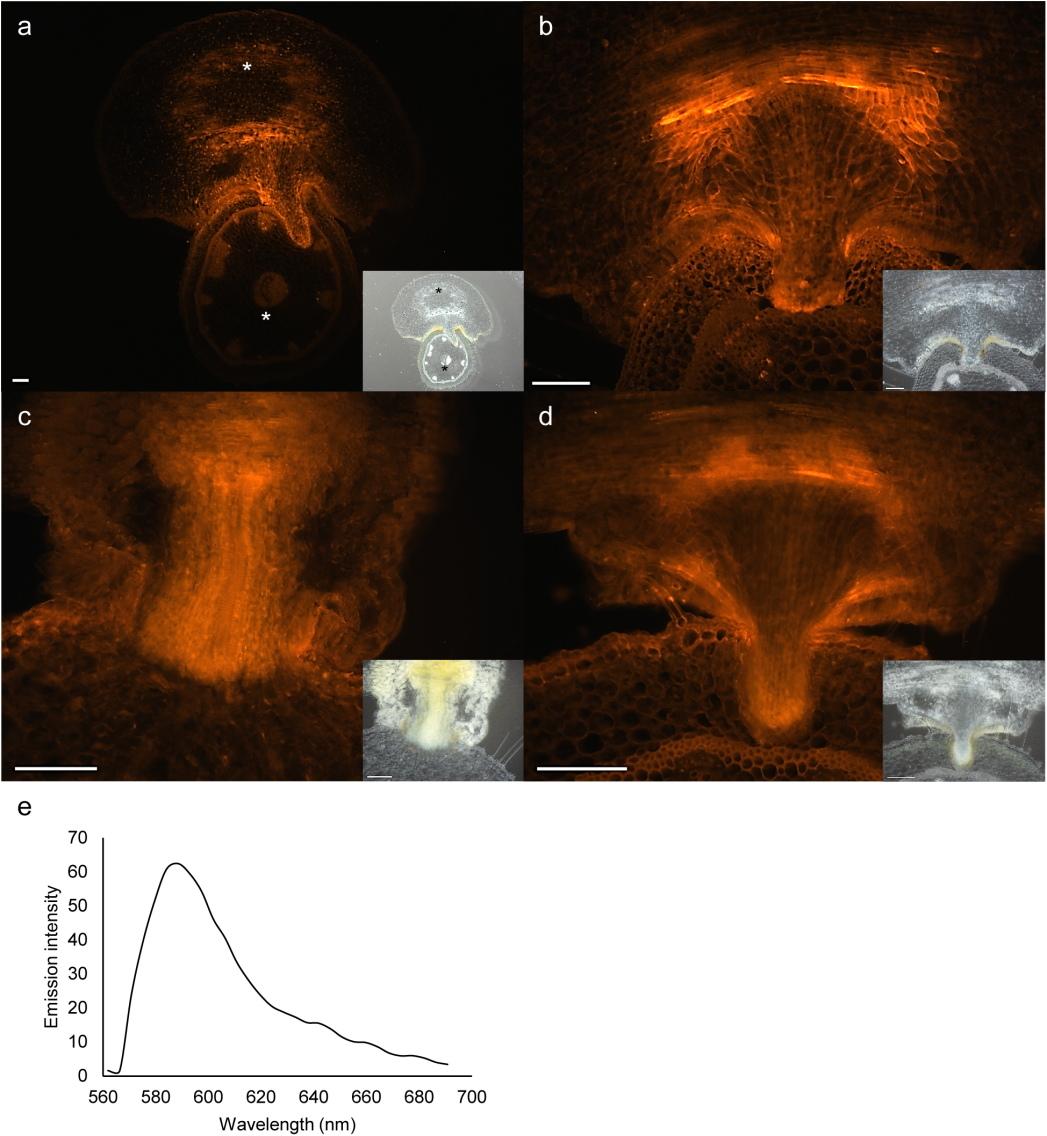
*In vivo* colocalization of XET activity and donor substrate in cross-sections of penetrating infection sites. Fluorescence reveals activity in cross-sections of (a, b) *C*. *reflexa*, (c) *C*. *campestris* or (d) *C*. *platyloba* infecting *P*. *zonale*. Asterisks in the overview picture of *C*. *reflexa* infecting *P*. *zonale* (a) indicate regions of low XET activity distal to the host-parasite interface. Brightfield images for morphological reference are shown as smaller inserts. Scale bars are 200 μm. (e) Wavelength-specific fluorescence emission intensities from cross-sections of infection sites, as analysed by confocal microscopy after incubation with XyGO-SR and subsequent washing steps.

The *in situ* localizations of XET-performing enzymes were determined by incubating cross-sections of *Cuscuta* infection sites with sulforhodamine-labelled xyloglucan oligosaccharides (XyGO-SR) and subsequently washing away unincorporated XyGO-SR. Colocalization of endogenous xyloglucan donor substrate and XET activity is indicated by fluorescence and was found in the upper part of the haustorium and at the host-parasite border of the endophytically growing haustorium of *C*. *reflexa*, *Cuscuta campestris* and *Cuscuta platyloba* (Fig 2). In contrast, the parts of host and parasite distal to the interface showed almost no activity (see asterisks in Fig 2A). To demonstrate that the observed fluorescence was a result of the incorporation of sulforhodamine-labelled xyloglucan, we recorded the emission spectrum of the fluorescence from infection sites incubated with XyGO-SR using a confocal microscope. A single emission peak around 590 nm (Fig 2E), which coincides with the excitation spectrum of sulforhodamine B (http://www.fluorophores.tugraz.at/substance/10), verified that the observed fluorescence was in fact originating from this compound. Moreover, infection sites without added XyGO-SR showed no autofluorescence with 400 ms exposure times (S1 Fig).

**Fig 3:**
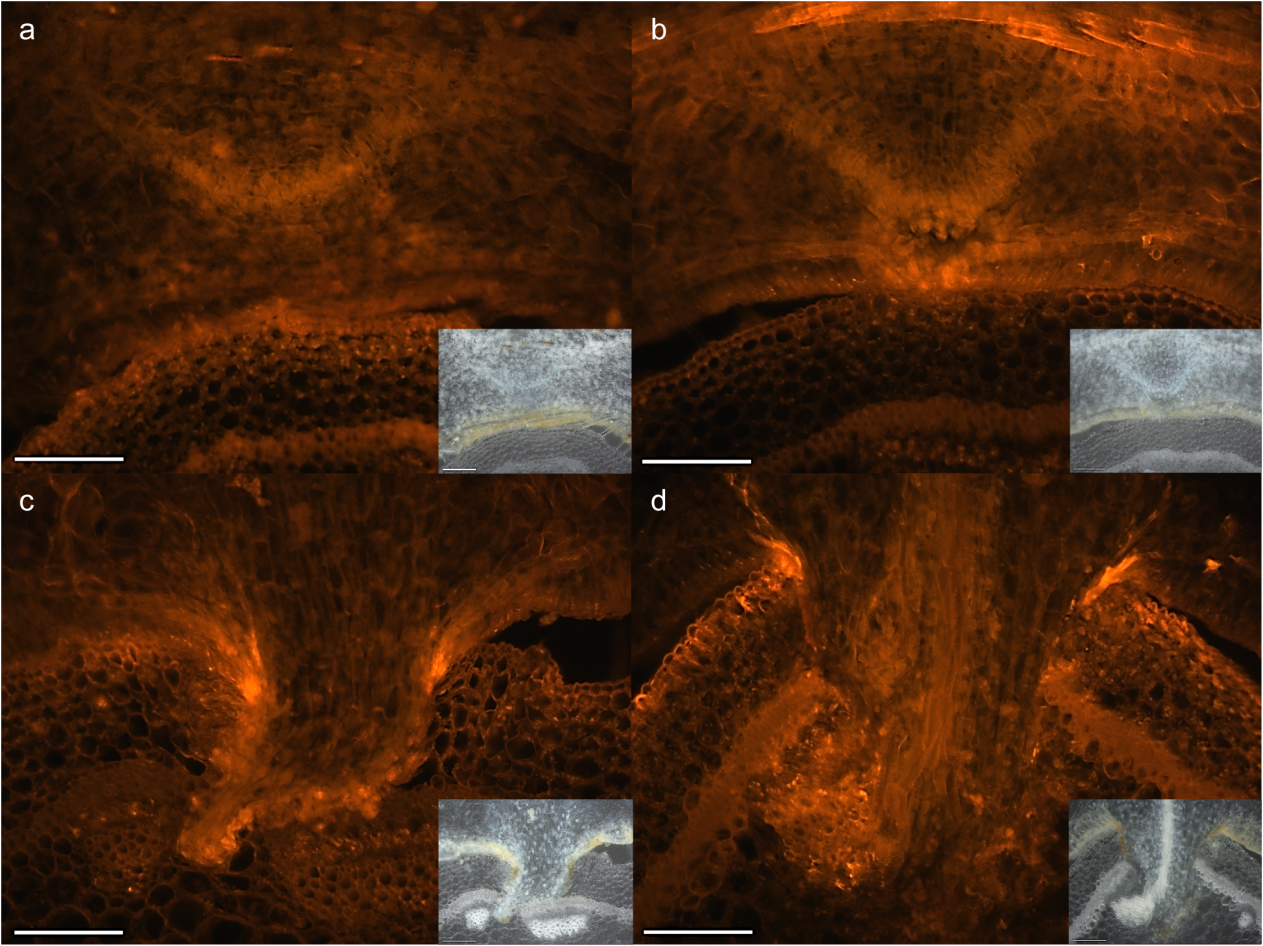
Infection stage-dependent *in situ* XET activity at the interface between *C*. *reflexa* and *P*. *zonale*. Colocalization of activity and donor substrate is indicated by fluorescence during (a) swelling, (b) early penetrating, (c) penetrating and (d) mature parasitization stages. For morphological reference, brightfield images are shown as smaller inserts. Scale bars are 200 μm.

High XET activity could also be seen at the haustorial tip just before it emerged from the parasite to start host invasion and as the infection organ grows through the host tissue (Fig 3B and 3C). In contrast to the stages where the haustorium is still developing and “on the move”, the fluorescence at the host-parasite interface was not observed in fully developed haustoria (Fig 3D).

Overall, the *in situ* observations corroborated the *in vitro* XET activity measurements and showed that the activity of XTHs is high in *Cuscuta* haustoria during the penetration of host tissue, but goes down once a mature feeding connection is established (Figs 1, 2 and 3).

### Haustoria of *C*. *reflexa* secrete enzymes performing XET

**Fig 4:**
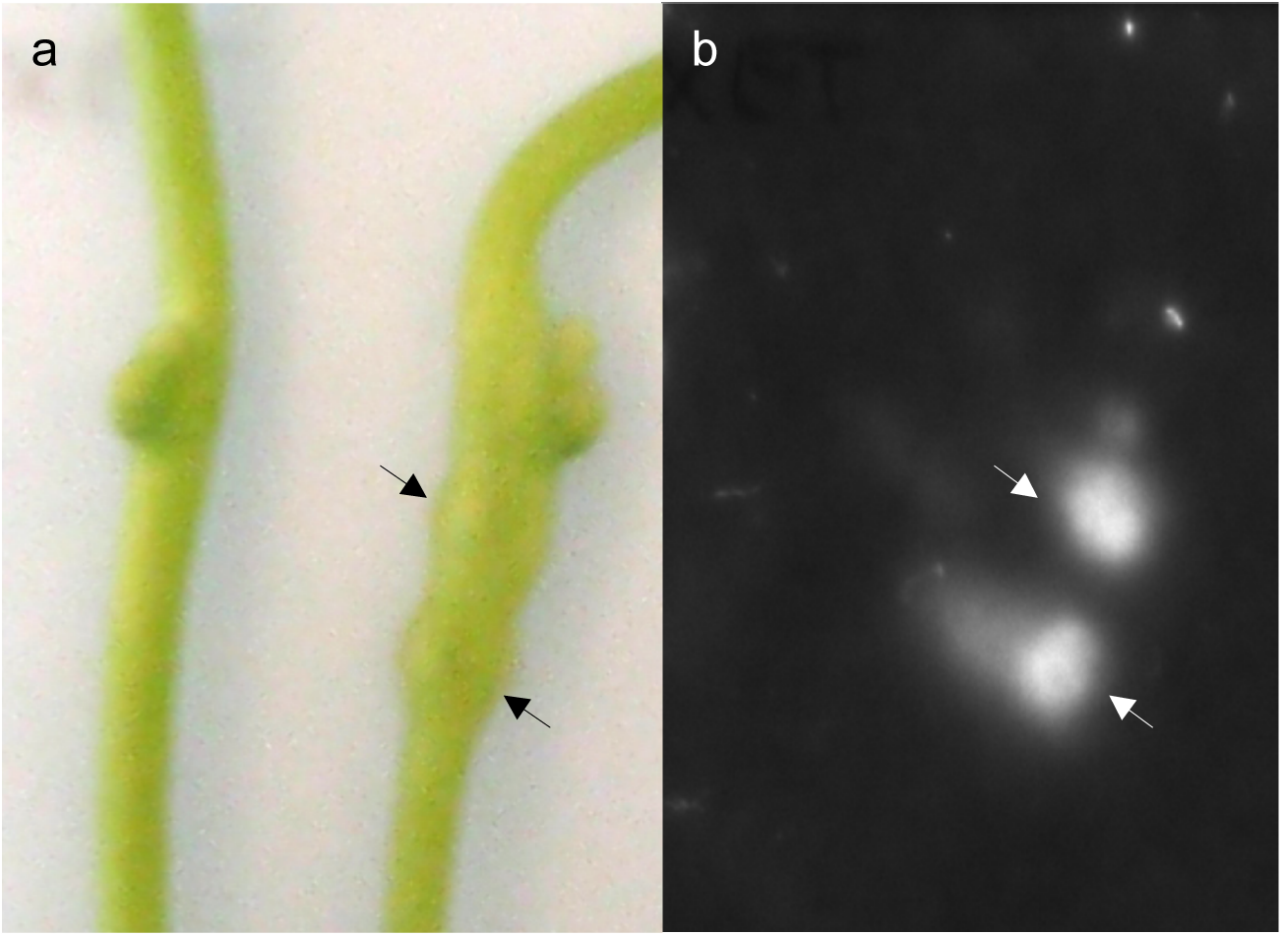
Secretion of XET-active enzymes from haustoria of *C*. *reflexa*. The localization of secreted XTHs is shown by fluorescence indicating the site-specific incorporation of XyGO-SR into xyloglucan. (a) Position of non-induced (left) and far-red light-induced (right) *Cuscuta* shoots on the XET test paper. (b) Fluorescence image of the XET test paper in (a) after incubation with parasite shoots and de-staining of background. Arrows point to single haustoria.

The *in situ* XET assays demonstrated endotransglucosylation of xyloglucan at the host-parasite interface. To further test if XET-active enzymes are being secreted from the growing infection organ, we developed an XET secretion test in which far-red light-induced intact haustoria were placed on XET test papers, enabling the detection of XTHs at the surface of the parasite. As haustoria strongly adhere to the plastic petri dishes used to deliver the tactile pressure needed for activation of haustoriogenesis, the removal of induced shoots resulted in undesired injuries to the haustorial tissues. To overcome this issue, four layers of 30 μm mesh nylon membrane were placed between the shoots and the plastic surface before far-red light irradiation. In this way, *Cuscuta* shoots with developed haustoria could be transferred onto XET test papers without the danger of tissue damage. While areas of the test paper exposed to shoots lacking haustoria did not produce any fluorescence, areas of the test paper exposed to shoots with developing haustoria showed a strong fluorescence signal where the haustoria emerged (Fig 4). No signal was detected when printing on control papers, verifying that the observed fluorescence is caused by the secretion of XET-performing enzymes, and not due to the discharge of endogenous fluorescent compounds.

### BB-R250 inhibits host infection

**Fig 5:**
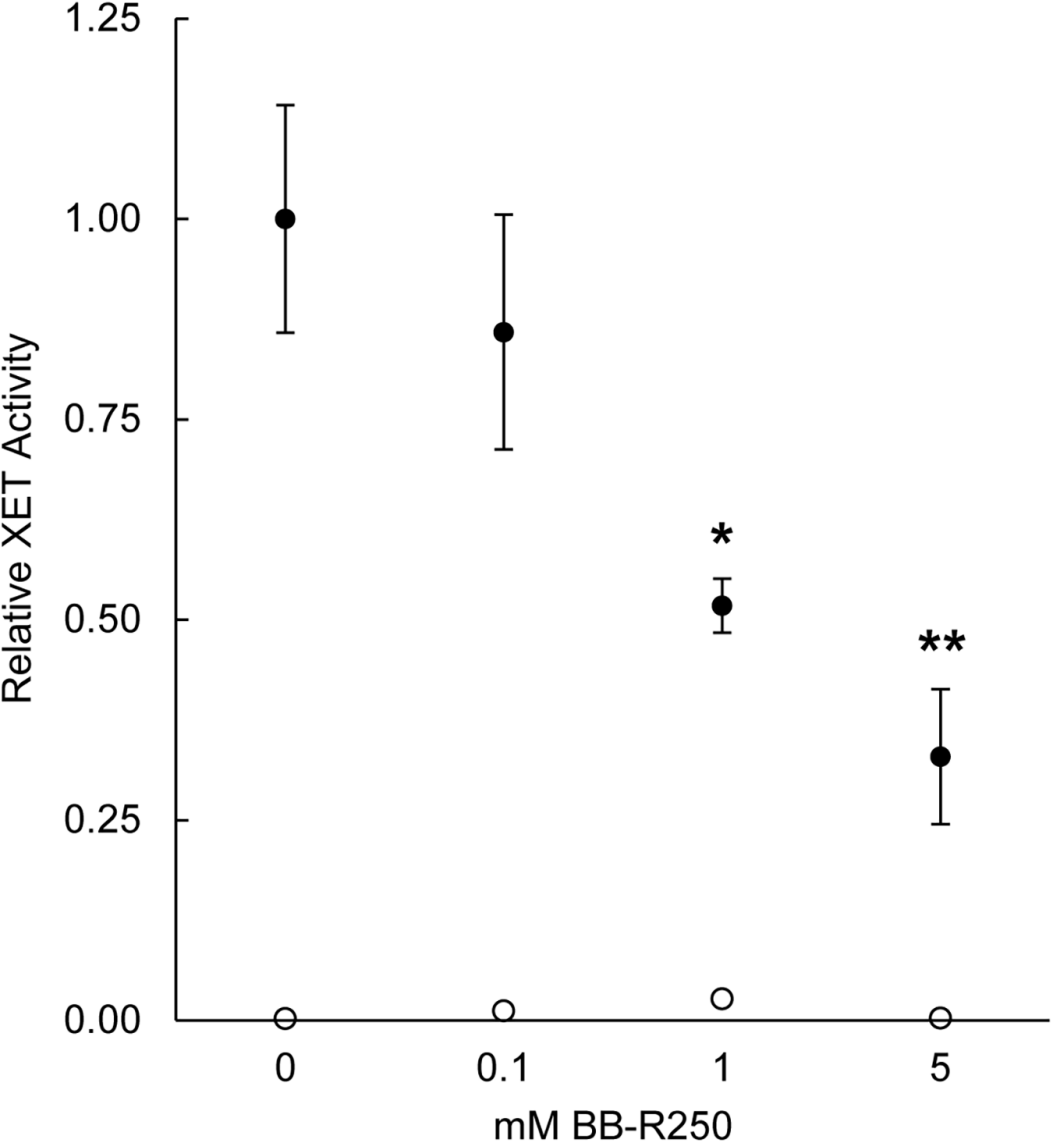
Effect of BB-R250 on XET activity in penetrating haustoria of *C*. *reflexa*. Activity levels were measured as the incorporation of XyGO-SR into xyloglucan by dot blotting on XET test papers. Fluorescence intensities (means ± standard deviation, n = 3 replicates) are presented in relation to the mean XET activity in extracts not added BB-R250 (set to 1). Statistically significant differences between the control without BB-R250 (0 mM) and the other samples were calculated by an unpaired, two-tailed *t*-test assuming unequal variances and are indicated by asterisks (*, P < 0.05 and **, P < 0.01). Empty circles indicate activity levels in boiled extracts.

The activity of *Cuscuta* XTHs at the host-parasite interface proposes that these cell wall-modifying enzymes play a role in the infection strategy of the parasite. Recently, a screen for inhibitors of xyloglucan endotransglucosylation reported the reduction of parsley XET activity by BB-R250 [19]. We found that BB-R250 also reduced the XET activity in enzyme extracts prepared from host-invading haustoria of *C*. *reflexa* and that the inhibitory effect was dependent on the concentration of BB-R250 (Fig 5).

**Fig 6:**
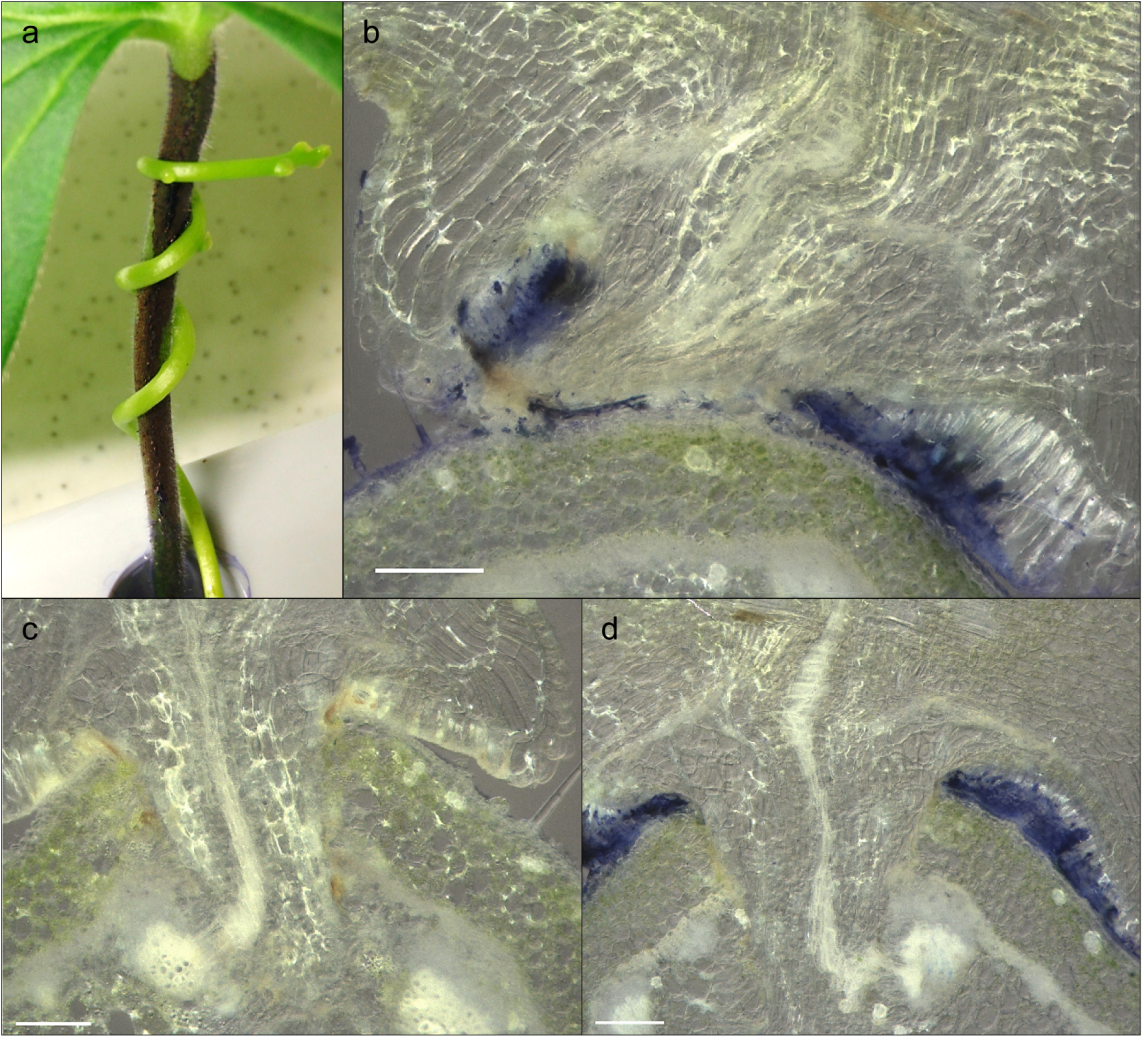
*C*. *reflexa* parasitization of BB-R250-coated and control *P*. *zonale* petioles. (a) Infection setup with the parasite twining around a BB-R250-coated petiole. (b, d) Cross-sections of *C*. *reflexa* infecting BB-R250-coated *P*. *zonale*. (c) Cross-section of *C*. *reflexa* infecting a petiole coated with 0.005% Poly-L-lysine without BB-R250 (control). Scale bars are 200 μm.

To test if the inhibitor also affects the establishment of an endophytic haustorium, *C*. *reflexa* was allowed to parasitize *P*. *zonale* petioles coated with BB-R250 (Fig 6A). The parasite showed no preference towards inhibitor-coated petioles or control petioles, readily starting to develop haustoria on any of them. However, sectioning and microscopical analysis of infection sites that had progressed to the mature stage (indicated by the continuation of apical tip growth 12–14 days after infection onset), revealed that approximately 1/3 of haustoria on 5 mM BB-R250-coated petioles had not been able to grow into the host tissue (Fig 6B and Table 1). During initial trials of the infection tests, petioles coated with 0.5 mM BB-R250 also displayed obstructed penetrations, but much less frequent. On the other hand, prevented haustorial invasions were never observed for any of the control petioles only coated with the solvent (Fig 6C and Table 1).

**Table 1:** Frequency of impeded *C*. *reflexa* infections on coated *P*. *zonale* petioles.

Coat concentration of BB-R250	Total number of sectioned haustoria	Number of prevented penetrations
0	**58**	**0**
5 mM	**70**	**23**

## Discussion

In a previous study, we found the expression of two *C*. *reflexa* genes, *Cr-XTH-1* and *Cr-XTH-2* that belong to a family of cell wall-modifying enzymes, the XTHs, to be specifically upregulated at the onset of haustorium development [6]. The XET activity of these enzymes modify the cell wall by cleaving xyloglucan chains and grafting them onto other chains, a process which has to a great extent been associated with the loosening of plant cell walls. When infecting a compatible plant, the *Cuscuta* haustorium appears to glide between the host cells [20, 21]. A loosening of host cell walls could make the tissue more pliable to such an intrusion. In fact, during its invasion of a host plant the plant-parasitic roundworm *Globodera rostochiensis* produces expansins, another type of wall-loosening proteins [22]. The specific localization of XET activity during *Cuscuta* host infection was determined using the *in situ* assay developed by Vissenberg *et al*. [23]. In addition to providing a better resolution than the XET tissue prints, this method contributes more information to what happens *in vivo*, as the generation of a fluorescence signal is dependent on the colocalization of endogenous xyloglucan donor substrate and XET-performing enzymes. Morphological studies have demonstrated that the haustorium originates from cortical cells, which in some species were just external of the pericycle [24, 25]. The initial cells divide and develop into a meristem from which the endophytic haustorium differentiates. At this onset of haustoriogenesis, the not yet emerged haustorium displayed XET activity at its growing tip (Fig 3A). As the infection organ of *C*. *reflexa* escapes its own tissue and begins to invade the tissue of *P*. *zonale*, the XET activity is increased and a distinct band of high XET activity was observed at the host-parasite interface on the periphery of the invading haustorium (Figs 1, 3B and 3C). The similar patterns of XET activity observed in host-invading haustoria of *C*. *campestris* and *C*. *platyloba* indicate that xyloglucan modification may be a general feature of the *Cuscuta* attack (Fig 2C and 2D). Modifications to xyloglucan are associated with plant growth. Accordingly, XET activity was also detected in the elongating stem region of the parasite (Fig 1). As the haustorium is a growing organ, the observed activity at the interface could be related to the loosening of parasite walls to allow cell expansion. On the other hand, the abundance of xyloglucan was earlier found to be reduced in infected host tissues as well as in the haustoria of *Cuscuta* [3, 26]. In addition to promoting the growth of the infection organ, modifications to xyloglucan could serve either one or both of two purposes: (i) The wall strength of the haustorial surface cells must be strictly regulated in order to withstand the physical pressure exerted on them during endophytic growth. In this case xyloglucan-modifiers would be expected to reside in the cell walls of the parasite. (ii) Xyloglucan modifications could ease the journey of the host-invading haustorium by loosening cell walls of the host. XET-performing enzymes were found to be secreted at those locations where haustoria were developing in apical shoot tips of *C*. *reflexa* (Fig 4), which implies that *Cuscuta* XTHs act on host cell walls. Moreover, no clear band of XET activity could be observed at the interface between parasite and host at the final stage of haustoriogenesis (Fig 3D), indicating that *C*. *reflexa* XTHs are most important during the penetrating stage of host infection.

Research on the hemicellulose xyloglucan and its modifiers is an active field and a few years back, Chormova *et al*. [19] presented a screen for small molecule inhibitors of XET, identifying BB-R250 as an inhibitor of this activity in parsley. Applied to *Cuscuta*, this compound was found to have two effects: it inhibited the level of XET in parasitic plant extracts in a dose-dependent manner (Fig 5) and it was able to reduce the number of successful host penetrations by about 30% (Fig 6 and Table 1). Unfortunately, the hydrophobic nature of the host surface made it impossible to completely cover the entire petiole with BB-R250, even when improving the adhesion by supplementing the solution with Poly-L-Lysine. In addition, 5 mM BB-R250 (the concentration used to coat petioles) did not completely inhibit the level of XET activity in *Cuscuta* enzyme extracts *in vitro* (Fig 5), which may explain why some haustoria were able to penetrate fully BB-R250-coated host surfaces (Fig 6D). Since Coomassie Brilliant Blue R250 binds to proteins in general, one cannot rule out that its association with proteins other than the XTHs influence its preventive effect on *Cuscuta* host infection. Infection tests with additional XET inhibitors or other protein stains could shed light on this matter. The *C*. *reflexa*-resistant *Solanum lycopersicum* was earlier shown to display higher abundances of the hemicelluloses xylan and mannan than did near-isogenic compatible *S*. *pennellii* introgression lines [3]. This is consistent with the parasite exploiting xyloglucan as an entryway into the host and could explain why *Cuscuta* in general does not parasitize grasses, which are reported to be xylan-rich and xyloglucan-poor [27]. We have here presented data indicating the essential function of *Cuscuta* XTHs during host infection and further showed that coating hosts with a small molecule inhibitor suppresses the tissue-invading capability of a *Cuscuta* species. It has earlier been shown that host plants sprayed with the inhibitory propeptide of a *Cuscuta*-encoded cysteine protease, were less prone to infection [28]. As parasitic plants can present a major threat to crop yields, these externally applied countermeasures offer exciting new possibilities to parasitic weed management.

## Materials and methods

### Plant growth and enzyme extraction

*Cuscuta* species (*C*. *reflexa*, *C*. *campestris* and *C*. *platyloba*) were originally obtained from the Botanical Garden of the University of Kiel and have been propagated vegetatively on the compatible host plant *P*. *zonale* in 24 h of light at 21°C at the Phytotron of UiT The Arctic University of Norway for the last 10 years. For the preparation of enzyme extracts, plant material was ground by mortar and pestle in ice-cold extraction buffer (50 mM sodium acetate (pH 5.5), 300 mM NaCl, 20 mM ascorbate, 10 mM CaCl_2_, 15% (v/v) glycerol, 3% (w/v) polyvinylpyrrolidone (PVP-40)) with a pinch of sea sand. One ml extraction buffer was used per 200 mg plant material. After 2 hours incubation on ice, the homogenate was centrifuged for 5 minutes at 12 000 *g* and the resulting supernatant stored at -20°C before further analyses. Protein concentrations were determined using the Bio-Rad Protein Assay according to the manufacture’s descriptions.

### XET dot blot

XET test papers coated with xyloglucan and XyGO-SR were prepared as described earlier [6]. Xyloglucan-coated papers without XyGO-SR were used as control papers. Dot blot assays were carried out essentially as described by Fry [29]. Enzyme extracts (1.5 μl volume) were spotted onto XET test paper at 4°C before the loaded paper was incubated between two sheets of acetate for 1 h at 21°C. When testing the inhibitory effect of BB-R250 on *C*. *reflexa* XET activity, an extract prepared from the parasitic tissue of penetrating infection sites was mixed with BB-R250 and incubated for 15 min at 21°C before spotting onto test papers as described above. The background was de-stained by washing the papers in ethanol: formic acid: water (1:1:1) for 2 h with gentle agitation followed by rinsing in distilled water. Images of dry papers were taken with a ChemiDoc MP Imaging System (Bio-Rad) using the Cy3 application with 400 ms exposure time. Relative levels of XET activity were calculated using global background-adjusted volumes. All dot blots were executed twice and mean values of both were used for calculations.

### *In vivo* colocalization of XET activity and donor substrate

Different stages of *Cuscuta* infection sites on *P*. *zonale* were collected based on morphological characteristics [6]. Cross-sections (100 μm thick) through the sites were prepared using a Leica VT1000E vibratome (Leica Biosystems, Nussloch GmbH, Nussloch, Germany) and provided confirmation regarding the stage of haustorium development. Localization of XET activity using endogenous xyloglucan as donor substrate was carried out as described by Vissenberg *et al*. [23] with minor modifications. Cross-sections were incubated in 5 μM XyGO-SR dissolved in 50 mM Na-acetate (pH 5.5), 300 mM NaCl for 1 h in the dark. Sections incubated in buffer without XyGO-SR served as controls. After 10 min washing in ethanol: formic acid: water (15:1:4), the sections were further de-stained overnight in 5% formic acid. Micrographs were taken with a SteREO Lumar V12 equipped with an AxioCam MRc5 camera and the Lumar 43 filter set (all from Carl Zeiss, Jena, Germany). Exposure times were between 300 and 520 ms. The fluorescence emission spectrum from 560 nm to 690 nm was recorded using a LSM780 confocal laser scanning microscope (Carl Zeiss).

### XET secretion test

Host-independent induction of haustoriogenesis by far-red light and tactile stimuli was carried out on apical shoots of *C*. *reflexa* as described earlier [6], but with one essential modification. Four layers of nylon membrane with 30 μm mesh size (Sefar Nitex 03-30/18, Sefar AG, Heiden AR, Switzerland) were put between the shoots and one petri dish. Six days after induction, shoots which had attached to the membranes and developed haustoria as well as non-induced shoots without haustoria were carefully transferred to pieces of XET test paper soaked in 50 mM Na-acetate, 300 mM NaCl (pH 5.5), ensuring that the membrane gets moistened in order to facilitate contact between the *Cuscuta* tissue and the test paper. Xyloglucan-coated papers without XyGO-SR were used as control papers. After incubation between two sheets of acetate for 1 hour, background de-staining and recording of the fluorescence on the papers were carried out as described for XET dot blots.

### Analysing the effect of BB-R250 on host infection

Detached petioles (5–10 cm) of *P*. *zonale* were dipped in 0.005% Poly-L-lysine with or without 5 mM BB-R250. The Poly-L-lysine increased the adhesion of the BB-R250 that otherwise rolls off the hydrophobic plant surface. After drying, the coated petioles were placed in water reservoirs. Apical shoot tips of *C*. *reflexa* were positioned in close proximity of the petioles to promote twining of the parasite around the host. Attachment rates were increased by far-red light irradiation for 30 minutes followed by overnight dark incubation. The next day, infection setups were committed to a 12 hours day/12 hours night regime. After 12–14 days, successful infections were scored by making cross-sections through all haustoria. Micrographs were taken with a SteREO Lumar V12 equipped with an AxioCam MRc5 camera.

## Supporting information

S1 Fig Negative controls of *in situ* XET assays. Cross-sections of *P*. *zonale* infected by (a, b) *C*. *reflexa*, (c, d) *C*. *campestris* or (e, f) *C*. *platyloba* were incubated in buffer without XyGO-SR before being washed as described for the labelled sections. Fluorescence micrographs were taken with 400 ms exposure times (a, c and e). To demonstrate the weak autofluorescence, the brightness was increased in same images (b, d and f, respectively). For morphological reference, brightfield images are shown as smaller inserts. Scale bars are 200 μm.
(TIF)
