# Supplementary figures and images for: Activity of xyloglucan endotransglucosylases/hydrolases suggests a role during host invasion by the parasitic plant Cuscuta reflexa
Source: PLoS One. 2017 Apr 27;12(4):e0176754. doi: 10.1371/journal.pone.0176754 (PMC5407826; doi:10.1371/journal.pone.0176754)

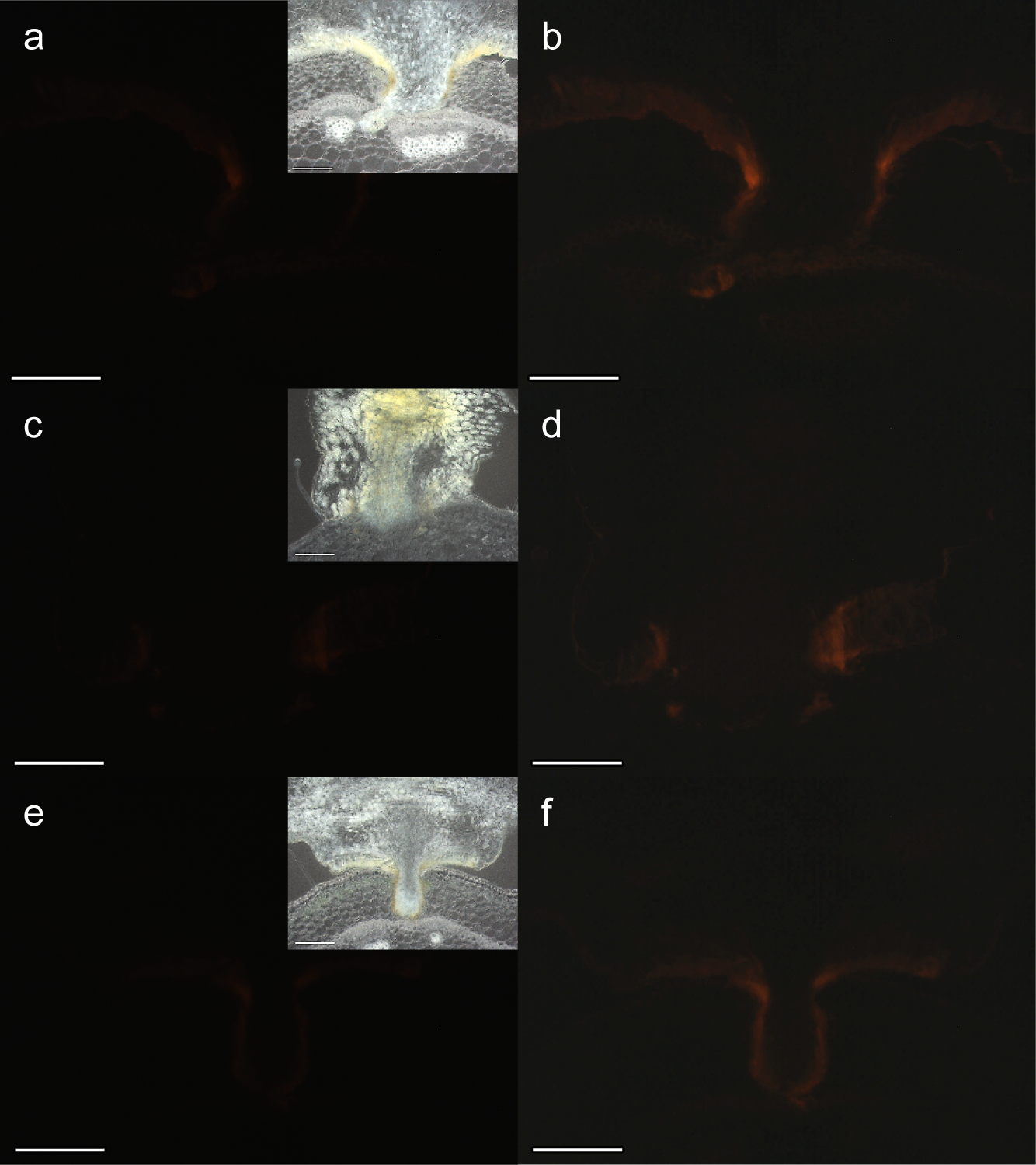

Supplement: S1 Fig — Cross-sections of P. zonale infected by (a, b) C. reflexa, (c, d) C. campestris or (e, f) C. platyloba were incubated in buffer without XyGO-SR before being washed as described for the labelled sections. Fluorescence micrographs were taken with 400 ms exposure times (a, c and e). To demonstrate the weak autofluorescence, the brightness was increased in same images (b, d and f, respectively). For morphological reference, brightfield images are shown as smaller inserts. Scale bars are 200 μm. (TIF) [file pone.0176754.s001.tif]
